# Short term outcome of myocarditis and pericarditis following COVID-19 vaccines: a cardiac magnetic resonance imaging study

**DOI:** 10.1007/s10554-023-02799-w

**Published:** 2023-03-13

**Authors:** Nicola Galea, Giulia Cundari, Emanuele Di Dedda, Cristina Chimenti, Giovanni Donato Aquaro, Andrea Barison, Riccardo Cau, Ernesto Di Cesare, Paolo Di Renzi, Antonio Esposito, Riccardo Faletti, Marco Gatti, Carlo Liguori, Luigi Lovato, Cesare Mantini, Caterina Beatrice Monti, Anna Palmisano, Silvia Pradella, Fabrizio Ricci, Luca Saba, Francesco Secchi, Carlo Catalano, Marco Francone

**Affiliations:** 1 Department of Radiological, Oncological and Pathological Sciences, Sapienza University of Rome, Viale Regina Elena 324, 00161 Rome, Italy; 2 Department of Experimental Medicine, “Sapienza” University of Rome, Viale Regina Elena 324, 00161 Rome, Italy; 3 Department of Biomedical Sciences, Humanitas University, via Rita Levi Montalcini 4, 20072 Pieve Emanuele, Milan, Italy; 4 IRCCS Humanitas Research Hospital, via Manzoni 56, 20089 Rozzano, Milan, Italy; 5 Department of Clinical, Internal, Anesthesiologist and Cardiovascular Sciences, Sapienza University of Rome, Viale Regina Elena 324, 00161 Rome, Italy; 6 Department of Surgical, Academic Radiology, Medical and Molecular Pathology and of Critical Area, University of Pisa, Pisa, Italy; 7 Fondazione Toscana Gabriele Monasterio, Pisa, Italy; 8 Department of Medical Sciences, Radiology Unit, University of Cagliari, Via Università 40, 09124 Cagliari, Italy; 9 Department of Life, Health and Environmental Sciences, University of L’Aquila, Piazzale Salvatore Tommasi 1, 67100 L’Aquila, Italy; 10 Radiology Division, Ospedale “San Giovanni Calibita” Fatebenefratelli Isola Tiberina, Via di Ponte Quattro Capi, 39, 00186 Rome, RM Italy; 11 Clinical and Experimental Radiology Unit, Experimental Imaging Center, IRCCS Ospedale San Raffaele, Milan, Italy; 12 Department of Surgical Sciences, Radiology Unit, University of Turin, Turin, Italy; 13 U.O.C. Diagnostica per Immagini. Ospedale del Mare - ASL NA1 Centro, Via Enrico Russo, 80147 Naples, Italy; 14 Department of Pediatric and Adult Cardio-Thoracovascular, Onchoematologic and Emergencies Radiology Unit, IRCCS Azienda Ospedaliero-Universitaria di Bologna, via Giuseppe Massarenti, 9, 40138 Bologna, BO Italy; 15 Department of Neuroscience, Imaging and Clinical Sciences, “G.d’Annunzio” University of Chieti-Pescara, Via dei Vestini, 33, 66100 Chieti, Italy; 16 Department of Biomedical Sciences for Health, Università Degli Studi Di Milano, Via Mangiagalli 31, 20133 Milano, Italy; 17 Department of Emergency Radiology, University Hospital Careggi, Florence, Italy; 18 Unit of Radiology, IRCCS Policlinico San Donato, Via Morandi 30, 20097 San Donato Milanese, Italy

**Keywords:** Magnetic resonance imaging, Myocarditis, Pericarditis, COVID-19, Drug interaction, Vaccine

## Abstract

To evaluate clinical and cardiac magnetic resonance (CMR) short-term follow-up (FU) in patients with vaccine-associated myocarditis, pericarditis or myo-pericarditis (VAMP) following COVID-19 vaccination. We retrospectively analyzed 44 patients (2 women, mean age: 31.7 ± 15.1 years) with clinical and CMR manifestations of VAMP, recruited from 13 large tertiary national centers. Inclusion criteria were troponin raise, interval between the last vaccination dose and onset of symptoms < 25 days and symptoms-to-CMR < 20 days. 29/44 patients underwent a short-term FU-CMR with a median time of 3.3 months. Ventricular volumes and CMR findings of cardiac injury were collected in all exams. Mean interval between the last vaccination dose and the onset of symptoms was 6.2 ± 5.6 days. 30/44 patients received a vaccination with Comirnaty, 12/44 with Spikevax, 1/44 with Vaxzevria and 1/44 with Janssen (18 after the first dose of vaccine, 20 after the second and 6 after the “booster” dose). Chest pain was the most frequent symptom (41/44), followed by fever (29/44), myalgia (17/44), dyspnea (13/44) and palpitations (11/44). At baseline, left ventricular ejection fraction (LV-EF) was reduced in 7 patients; wall motion abnormalities have been detected in 10. Myocardial edema was found in 35 (79.5%) and LGE in 40 (90.9%) patients. Clinical FU revealed symptoms persistence in 8/44 patients. At FU-CMR, LV-EF was reduced only in 2 patients, myocardial edema was present in 8/29 patients and LGE in 26/29. VAMPs appear to have a mild clinical presentation, with self-limiting course and resolution of CMR signs of active inflammation at short-term follow-up in most of the cases.

**Supplementary Information:**

The online version contains supplementary material available at 10.1007/s10554-023-02799-w.

## Introduction

Since late 2020, various types of vaccines with different vectors and mechanisms of action have been authorized and administered for immunization against COVID-19 and in about one and a half years, approximately 5.28 billion people received at least one dose of vaccine [1].

These vaccines demonstrated a strong safety profile, with extremely low rate of side effects and complications. Those include rare cases of vaccine-associated myocarditis or pericarditis (VAMP), mainly affecting young male individuals with higher risk within the first weeks from the second dose and using mRNA-based vaccines (source: https://www.cdc.gov/coronavirus/2019-ncov/vaccines/safety/myocarditis.html).

The real frequency and pathogenetic mechanisms underlying the myocardial and pericardial inflammation following vaccination are still poorly understood and highly debated [2–4].

In a recent meta-analysis, pooled incidence of myocarditis following COVID-19 vaccine on an overall cohort of 17,704,413 subjects was 35/million over an observation period of about two years, with only a single fatal case [4]. Although not uncommon, VAMPs are less frequent than COVID-19 related myocarditis [5].

Case series of VAMPs reported a generally favorable clinical course with spontaneous resolution of symptoms during the first weeks and a very low rate of severe or life-threatening forms, similarly to viral myocarditis or pericarditis [6–9]. However, in some subjects myocardial inflammation could persist after months, requiring closer follow-up and specific therapies.

Characterizing clinical evolution of VAMPs and their correlation with imaging features of inflammation, would provide further insights into the clinical significance and prognostic determinants of this rare post-vaccine complication. This appears particularly necessary in order to answer the pressing request of data about the real safety profile of the vaccination campaign by public opinion.

Cardiac Magnetic Resonance (CMR) is widely considered as the reference non-invasive diagnostic option to confirm the diagnosis of VAMPs and to drive clinical decision-making and follow-up.

Our purpose was therefore to explore baseline and follow-up clinical and CMR features in a cohort of individuals with VAMPs recruited from a multicenter consortium of 13 national tertiary hospitals.

## Methods

### Study population

Our target population was retrospectively selected from a cohort of individuals presenting with cardiac symptoms, laboratory and imaging findings suggestive for an acute myocardial damage, within 25 days from COVID-19 vaccine injection, from 13 large tertiary Italian hospitals in the period from March 2021 to February 2022.

Our inclusion criteria were the following: (1) cardiac symptoms and/or ECG abnormalities, (2) troponin serum level increase, (3) evidence of active cardiac injury at a CMR examination performed within 20 days from symptom onset and 4) clinical and/or CMR follow-up of at least 30 days.

All patients had a confirmed diagnosis of myocarditis, pericarditis or myopericarditis as proposed by the Center for Disease Control and prevention [10, 11].

Patients with known pre-existing chronic conditions associated with myocardial inflammation (e.g. chronic myocarditis, rheumatic or autoimmune diseases, vasculitis) were excluded from the evaluation.

The study was approved by the institutional review board of the leading center. Informed consent was obtained from all the patients.

### CMR protocols and findings

All demographic, clinical, laboratory and CMR data at baseline and short-term follow-up from all centers were collected, anonymized and analyzed by Sapienza University group.

Troponin serum level was considered increased if  > 99th percentile than the normal range of each local laboratory standard.

All CMR protocols from different centers (Supplementary Table) included T2-weighted images, cine images and late gadolinium enhancement (LGE) sequences acquired after contrast media administration. In ten centers, T1 and/or T2 mapping sequences were also acquired. CMR images were evaluated by local radiologists with various years of experience in cardiac imaging (minimum 8 years). Differential diagnoses with alternative causes of myocardial injury were at the discretion of local physicians based on local assessments.

Left and right ventricular volumes and CMR features of tissue damage (presence of myocardial edema, LGE areas and abnormal myocardial T1 or T2 values) were collected in all patients. Left ventricular (LV) systolic function was categorized as normal if left ventricular ejection fraction (LVEF) was ≥ 50%; mildly decreased systolic function was defined with LVEF between 40 and 50%, moderately decreased if 30–40%, and severely decreased if  < 30% [12].

According to the revised Lake Louise Criteria [13], active myocardial inflammation was defined by the presence of T1 criterion (LGE or native T1 increase or extracellular volume increase) and T2 criterion (edema in T2-weighted images or T2 ratio > 2 or T2 mapping increase). LGE and edema were visually assessed and categorized as “present” or “not present”; on both images, the distribution of LGE or edema was classified as subendocardial, mid-wall, subepicardial or transmural; the extent of the LGE was quantified as the number of segments involved according to the 16 segments model. Regional or global increase in T1 and T2 mapping values were defined when  > 2 standard deviation (SD) above the local reference values, which vary for specific MR sequence/equipment, calculated from the local group based on a sample of healthy controls, as suggested by consensus document [14].

CMR signs of pericarditis, defined as thickening and enhancement of the pericardial layers with or without pericardial effusion, have been detected [15].

Clinical follow-up data were acquired with electronic medical records, follow-up visits or phone interviews.

### Statistical analysis

Continuous variables were expressed as mean ± standard deviation (if normality could be assumed using the Shapiro-Wilks test) or median values with range. Independent variables were compared with unpaired t-test. Categorical variables, reported as counts and percentages, were arranged in cross-correlation tables and studied with the χ2 test or Fisher’s exact test. All the tests were 2-tailed, and only p values < 0.05 were considered statistically significant. Analysis was performed using SPSS software version 26.0 (IBM).

## Results

### Population and vaccination

**Fig. 1:**
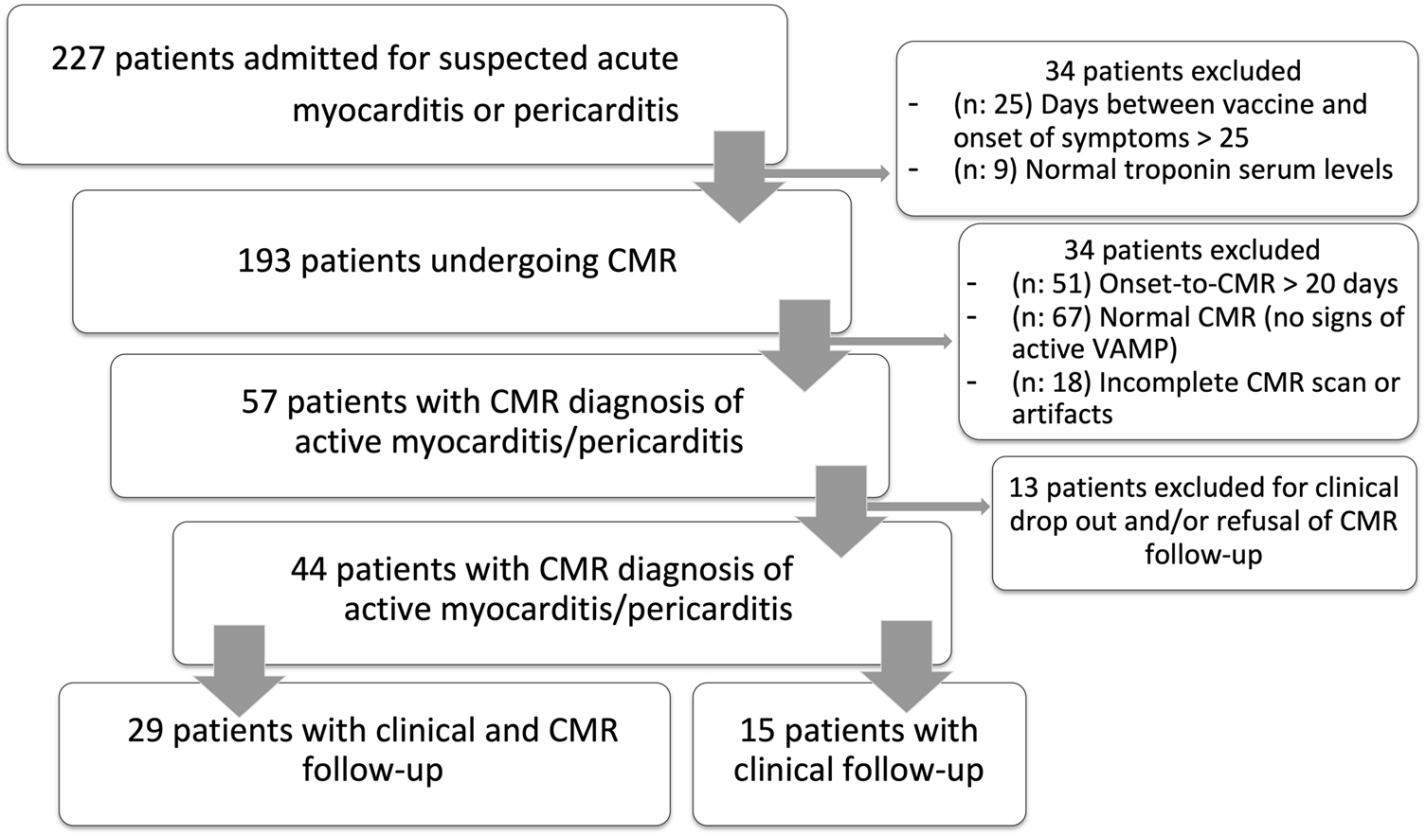
Flow-chart of patient population selection. On the right, the number of subjects excluded according to inclusion and exclusion criteria

A total of 44 patients were finally included in the study [females were 2 (4.5%)], with a mean age of 31.7 ± 15.1 years old. All the patients were aged ≥ 15 years old and 29 (65.9%) were < 35 years old. Patient selection flow-chart is reported on Fig. 1.

**Fig. 2:**
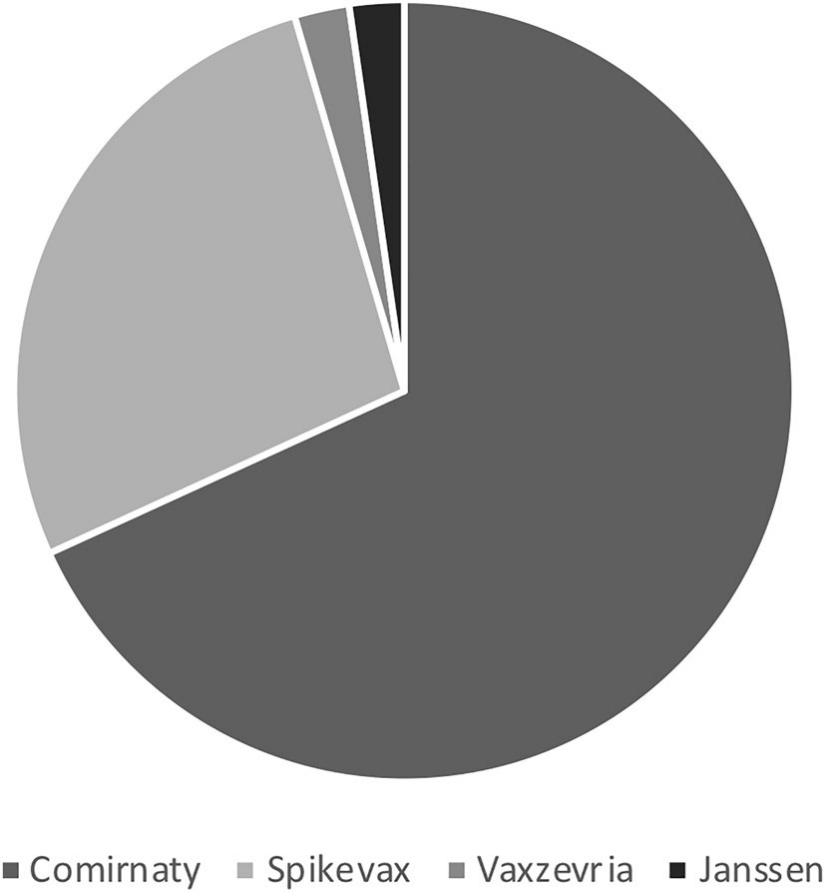
Pie chart illustrating the types of vaccine administered before the onset of symptoms

Mean time from the last vaccination dose to the onset of symptoms was 6.2 ± 5.6 days. The majority of the subjects included in the analysis received a vaccination with mRNA vaccine (Fig. 2) and predominantly after the second dose (Table 1).

**Table 1:** General characteristics of study population. Data are reported as number of patients and percentages in brackets, unless otherwise indicated

Variable	Values	
Patient population	44	
Age, years (mean ± SD)	31.7 ± 15.1	
Sex, female	2 (4.5)	
Previous COVID-19 infection	2 (4.5)	
Vaccine		
Comirnaty	30 (68.1)	
Spikevax	12 (27.2)	
Vaxzevria	1 (2.3)	
Janssen	1 (2.3)	
Last dose of vaccine before symptoms		
First dose	18 (40.9)	
Second dose	20 (45.5)	
Booster dose	6 (13.6)	
Days from last dose to symptom onset (mean ± SD)	6.2 ± 5.6	
Cardiovascular risk factors		
Smoker	14 (31.8)	
Hyperlipidemia	5 (11.4)	
Hypertension	4 (9)	
Prior AMI	1 (2.3)	
Stroke	0	
Clinical features	Onset	Follow-up
Fever	29 (65.9)	0
Chest pain	41 (93.2)	5 (11.4)
Palpitations	11 (25)	2 (4.5)
Myalgia	17 (38.6)	2 (4.5)
Dyspnea	13 (29.5)	3 (6.8)
Troponin elevation	44 (100)	n/a
ECG anomalies	30 (68.1)	5 (11.4)
Major ventricular arrhythmias	0	0
Cardiac arrest	0	0

Clinical features	Onset	Follow-up
Fever	29 (65.9)	0
Chest pain	41 (93.2)	5 (11.4)
Palpitations	11 (25)	2 (4.5)
Myalgia	17 (38.6)	2 (4.5)
Dyspnea	13 (29.5)	3 (6.8)
Troponin elevation	44 (100)	n/a
ECG anomalies	30 (68.1)	5 (11.4)
Major ventricular arrhythmias	0	0
Cardiac arrest	0	0

*SD* standard deviation; *AMI* acute myocardial infarction

Two patients had a history of previous Sars-CoV-2 infection (previous Sars-CoV-2 nasopharyngeal swab RT-PCR testing positivity), both at least six months earlier than the vaccine administration. One patient had a previous acute myocardial infarction. None presented with known valvular pathologies, stroke or tumors.

### Baseline clinical and CMR features

Clinical, laboratory and CMR data are reported in Tables 1 and 2.

**Ta﻿ble 2:** CMR features of study population at baseline and follow-up. Data are reported as number of patients and percentages in brackets, unless otherwise indicated

Variable	Values		
Days from symptom onset to CMR (mean ± SD)	6.8 ± 4		
Days from baseline CMR to follow-up (mean ± SD)	130.7 ± 88.3		
	CMR baseline	CMR follow-up	*p* value
LV EDV/BSA, ml/m^2^ (mean ± SD)	83.5 ± 18.8	77.5 ± 16.2	0.168
LV EF, % (mean ± SD)	58.6 ± 8.8	60.1 ± 6.9	0.453
LV EF: > 50%	37/44 (84.1)	27/29 (93.1)	0.398
LV EF: 40–50%	5/44 (11.4)	2/29 (6.9)	
LV EF: 30–40%	2/44 (4.5)	0	
LV EF: < 30%	0	0	
LV MASS/BSA, g/m^2^ (mean	61.9 ± 11.7	61.6 ± 14.7	0.937
RV EDV/BSA, ml/m^2^ (mean ± SD)	83.1 ± 14.2	81 ± 16.1	0.572
RV EF, % (mean ± SD)	56.6 ± 6.9	56.7 ± 8.3	0.959
RV EF: > 50%	36/44 (81.8)	24/29 (82.7)	0.918
RV EF: 40–50%	8/44 (18.2)	5/29 (17.2)	
RV EF: 30–40%	0	0	
RV EF: < 30%	0	0	
Wall motion abnormalities	10/44 (22.7)	4/29 (13.8)	0.383
Maximum wall thickness, mm (mean ± SD)	9.5 ± 1.7	9.7 ± 1.6	0.700
Edema	35/44 (79.5)	4/29 (13.8)	** < *0.001***
Subepi-mesocardial	28/35 (80)	4/4 (100)	
Transmural	7/35 (20)	0	
LGE	40/44 (90.9)	26/29 (89.6)	0. 859
Subepi-mesocardial	39/40 (97.5)	26/26 (100)	
Transmural	1/40 (2.5)	0	
LGE + Segments	3.39 ± 2.7	2.0 ± 1.7	***0.016***
Increased nT1 values	24/35 (68.6)	6/16 (37.5)	***0.004***
Increased ECV values	12/24 (50)	1/11 (9)	***0.033***
Increased T2 values	28/34 (82.4)	8/21 (38)	***0.003***
Pericardial effusion	17/44 (38.6)	4/29 (13.8)	***0.018***
Diagnosis			
Myocarditis	28/44 (63.6)	7/29 (24.1)	
Pericarditis	3/44 (6.8)	0	
Myo-pericarditis	13/44 (29.5)	1/29 (3.4)	

*LV* left ventricle; *EDV* end diastolic volume; *BSA* body surface area; *EF* ejection fraction; *RV* right ventricle; *LGE* late gadolinium enhancement; *SD* standard deviation; *EDV* extracellular volume; *CMR* cardiac magnetic resonance. Increase of regional or global myocardial *n*T1, ECV and T2 values. *P* values in bold for *p* < 0.05

Chest pain was present in 41 patients (93.2%) who referred to the Emergency Department. Other symptoms included fever (29, 65.9%), myalgia (17, 38.6%), dyspnea (13, 29.5%) and palpitations (11, 25%). ECG anomalies (including ST segment elevation or depression, T-wave inversion, left or right bundle branch block and repolarization abnormalities) were found in 30 (68.1%).

Mean onset-to-CMR time was 6.8 ± 4 days. At CMR acquired during the acute phase, 5 (11.4%) patients presented with mild reduction of LVEF, whereas two patients (4.5%) had moderate LV function impairment and right ventricular systolic function was mildly reduced in 8 (18.2%) patients.

Regarding tissue characterization, edema was found in 35 (79.5%) and LGE in 40 (90.9%), both with a predominant subepicardial or mid-wall distribution pattern (80 and 97.5%, respectively), predominantly located in the mid-basal infero-lateral wall of the LV. Myocardial edema pattern was transmural in 7/35 (20%), whereas LGE was transmural in 1/40 (2.5%).

Mapping sequences were available in 35/44 patients for T1 and 34/44 patients for T2. T1 and T2 maps revealed regional or global increase of native T1 mapping in 24/35 (68.6%) patients and of T2 mapping in 28/34 (82.4%).

**Fig. 3:**
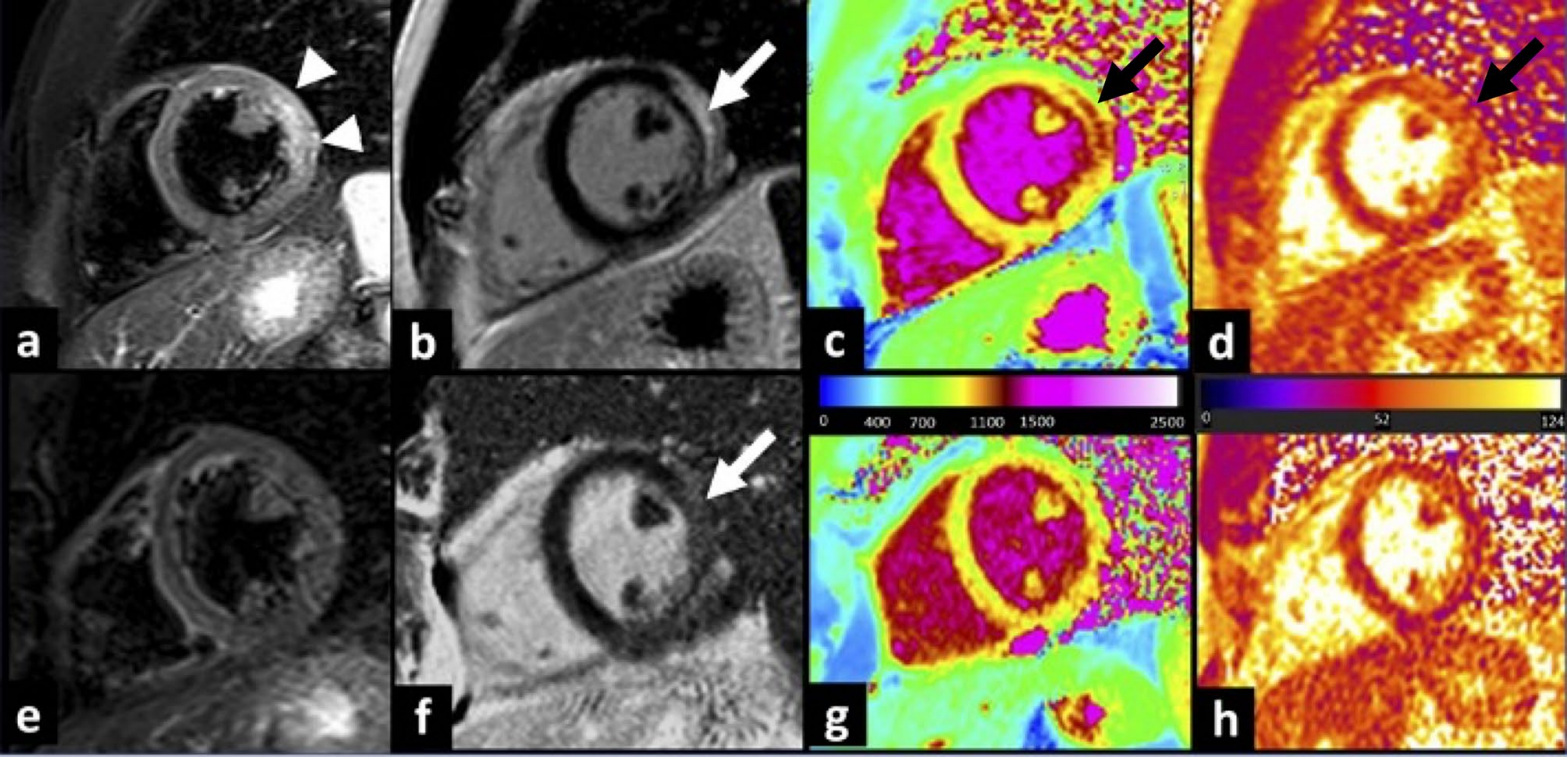
Vaccine-associated myocarditis. T2-weighted images (**a**) revealed the presence of myocardial edema located in the lateral wall of the left ventricle (arrowhead). A subepicardial LGE stria was evidenced on the same myocardial segments (**b**, white arrow). Those findings were confirmed by increased T1 (**c**) and T2 (**d**) mapping values on the lateral wall. At 117 days follow-up, CMR revealed a resolution of myocardial edema (**e**), with a reduction in LGE extension (**f**). Mapping sequences showed a decrease in T1 mapping values (**g**) and T2 values (**h**) that returned within the normal range

**Fig. 4:**
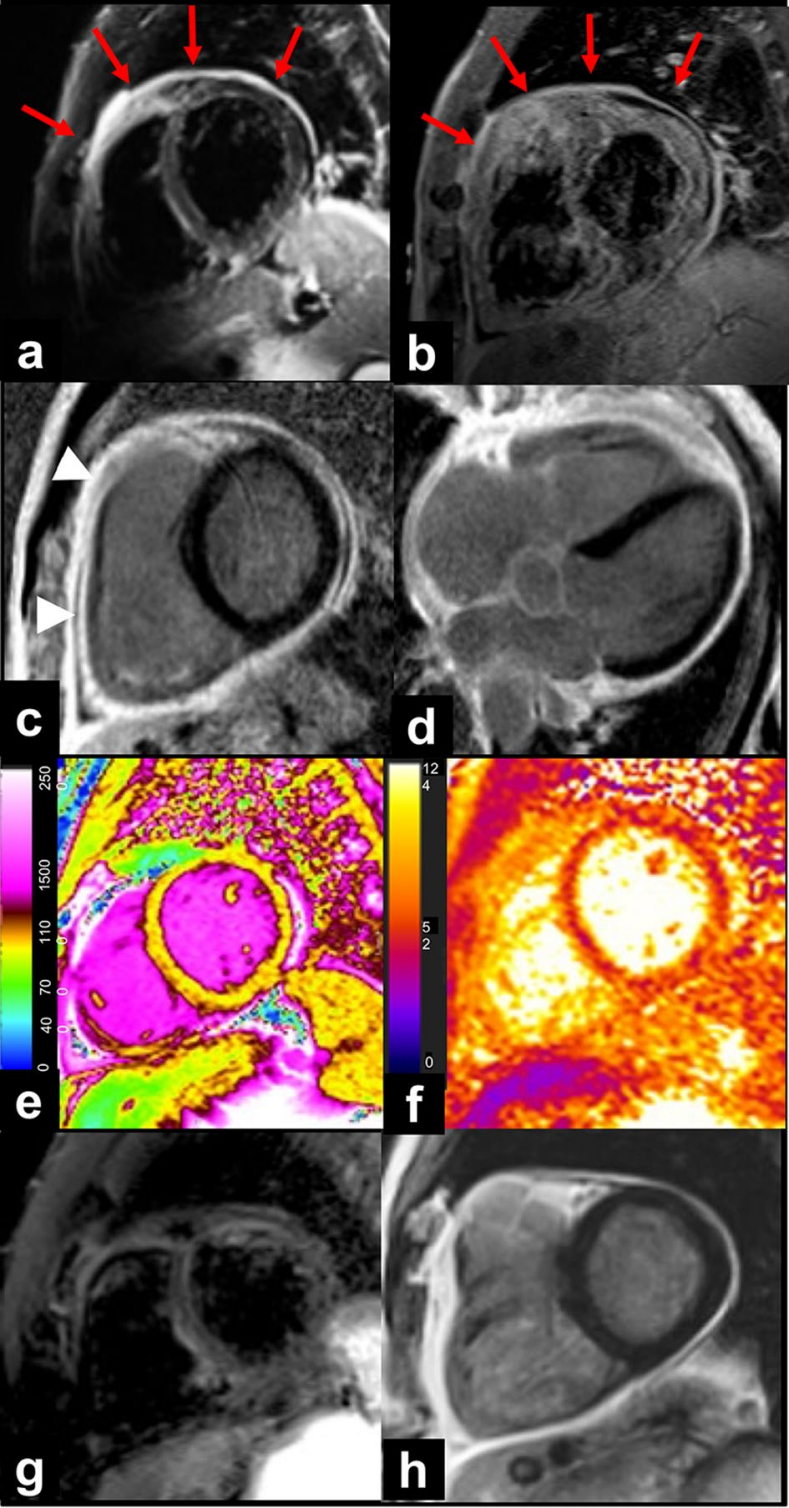
Vaccine-associated pericarditis. 57-year-old male admitted to ED for chest pain, fatigue and dyspnea after 8 days following second dose of Comirnaty vaccine. The first CMR (**a**–**f**), performed for the clinical suspicion of myocarditis, showed edematous thickening and enhancement of pericardial layers (red arrows) respectively on STIR T2-weighted (**a**) and fat-suppressed turbo spin echo T1-weighted (**b**) images. Absence of myocardial injury and pericardial enhancement (arrowheads) has been detected on late gadolinium enhanced images acquired on short axis (**c**) and horizontal long axis (**d**) view. Myocardial native T1 (**e**) and T2 (**f**) values were within normal range on corresponding maps. At 13-weeks follow-up CMR, there were neither pericardial fluid nor edema on STIR images (**g**) nor myocardial LGE areas (**h**)

Diagnosis at discharge of active myocarditis was reached in 28 (63.6%) patients (Fig. 3); myo-pericarditis in 13 (29.5%) and pericarditis in 3 (6.8%) (Fig. 4).

### Clinical and CMR follow-up

Clinical follow-up (FU) revealed absence of major events in all subjects and resolution of symptoms in most of them (82%). Persistent chest pain, myalgia, palpitations and dyspnea have been detected in 5 (11.4%), 2 (4.5%), 2 (4.5%) and 3 (6.8%) cases, respectively. ECG anomalies (repolarization abnormalities and left or right bundle branch block) were found in 5 (11.4%); none of the patients showed fever.

**Fig. 5:**
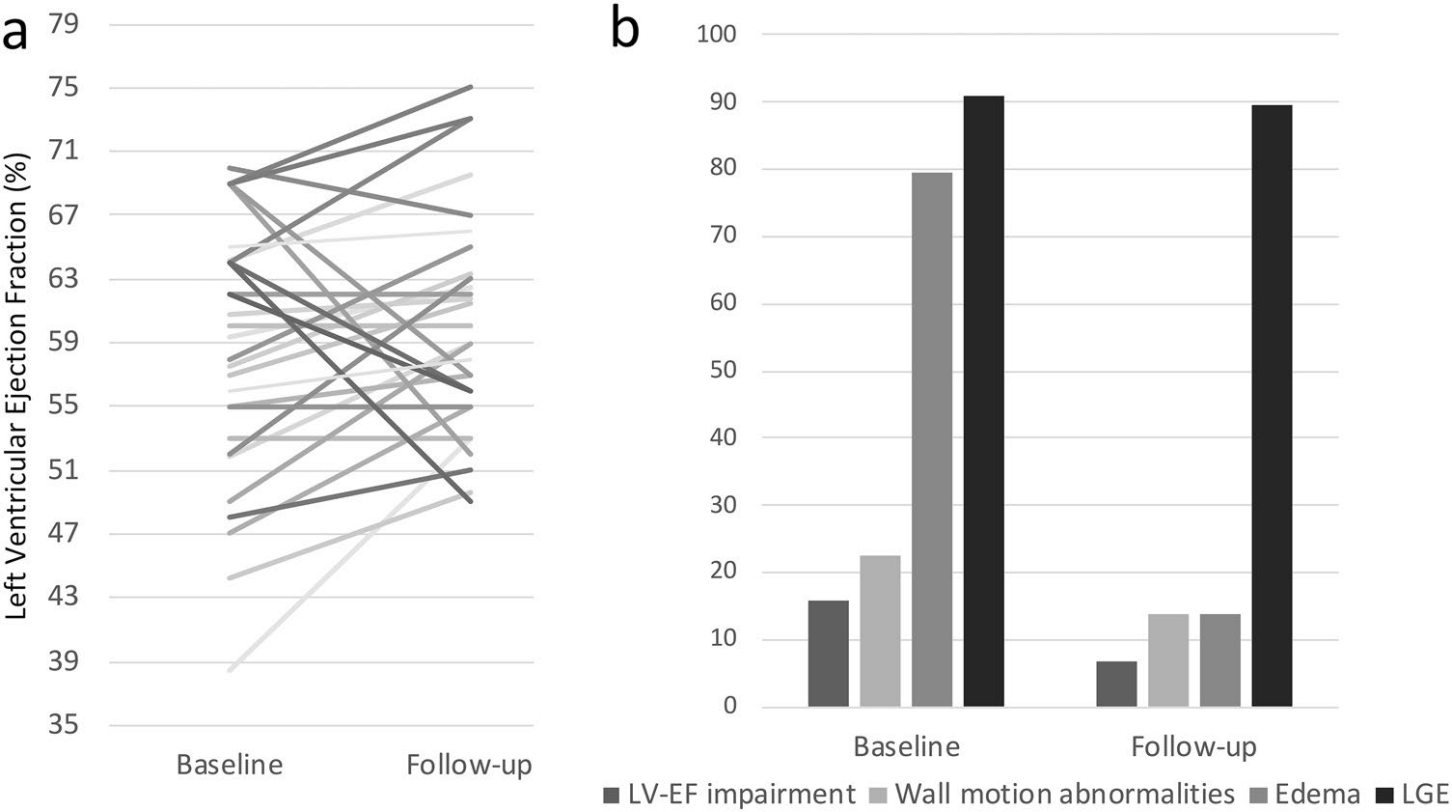
On panel A, a representation of left ventricular ejection fraction (LVEF) for each patient, at baseline and follow-up. Panel B illustrates population percentages with LVEF impairment, wall motion abnormalities, myocardial edema and LGE both at baseline and follow-up

FU-CMR was available in 29 patients and revealed recovery of left ventricular function in 5 patients with reduced LVEF at baseline CMR (Fig. 5a) and persisting mild LVEF reduction in 2/29 (6.9%).

Mapping sequences were available in 16/29 patients for T1 and 21/29 patients for T2. Persistent signs of active myocardial inflammation were present in 8/29 (27.6%) patients, who showed an increase in T2 mapping values (8/29) and/or hyperintense areas on T2-weighted images (4/29). LGE was found in 26/29 (89.6%) patients, with a typical subepicardial or mid-layer distribution (Fig. 5b). Native T1 values were increased in 6 cases. Residual thickening and hyperintensity of the pericardial layers were found in just one patient, whereas pericardial effusion in 4/29 (13.8%). At CMR follow-up, signs of myocardial inflammatory activity on T2-weighted and mapping sequences or pericardial inflammation were significantly reduced (p = 0.033–0.001). Although there was no significant modification of LGE occurrence, the mean number of LGE + segments was reduced at follow-up (p: 0.016). Finally, no significant correlation was found between the presence of symptoms at clinical FU and the persistence of edema revealed by FU-CMR.

## Discussion

Our study describes clinical and CMR characteristics of 44 patients, recruited from 13 tertiary national reference centers, that received a diagnosis of acute myocarditis, pericarditis or myo-pericarditis temporally related to COVID-19 vaccination, over a three-month follow-up period. To the best of our knowledge, our multicentric case collection included the largest patient cohort with CMR follow-up data reported in literature up to date.

Although complications related to COVID-19 vaccines are not fully explored, given their recent approval, VAMPs have been already extensively described as isolated cases or case series, especially when using mRNA vaccines [3, 7, 8, 16–18]. They represent a public safety concern, emphasized by the constant media attention to the worldwide massive vaccination campaign, and long-term implications need to be clarified.

As often pointed out, it should be specified that the temporal proximity between symptoms onset and vaccine inoculation is not per se sufficient to prove a causal link, and results of observational study on the general population should be considered cautiously. However, much evidences suggest that the vaccine may play a role as a trigger or a contributing cause of pericardial or myocardial inflammation in vaccinated patients [19–22]. Theoretically, the immune response induced by vaccination could also re-activate myocardial inflammation in subjects with recurrent myocarditis or chronic systemic inflammatory disease.

The first main finding emerged from our results is that VAMPs generally consist in a clinically uneventful syndrome with mild presentation and favorable outcome at follow-up. Second, CMR showed left ventricular functional recovery at short term follow-up in most of the cases, and significant reduction of signs of inflammation on CMR, which persisted in almost a quarter of the study population.

Our study population showed signs or symptoms of cardiac involvement with an average of 6.2 ± 5.6 days from the last dose of vaccine. According to the literature, the time interval between vaccination and cardiac symptoms may vary between 0 and 179 days [23], with most of the cases occurring within 7 days and a median time onset of 3 days [24]. Also, the first peak of clinical manifestation (within 1–3 days post vaccination) seems to be more associated with acute myocarditis; the second (between 15- and 30-days post-vaccination) with acute pericarditis [23].

In line with other reports [3, 7–9, 16–19], the most of our cases occurred using RNA-based vaccines. Anastassopoulou et al. [23] found Janssen and Vaxzevria to be more associated with cases of acute pericarditis, but in our cohort those vaccines both lead to myocarditis.

Furthermore, 95.5% of our patients were male and 65.9% were < 35 years old. The higher prevalence of acute myocardial injury among males has been attributed to the effects of sexual hormones on the immune response [19]: testosterone seems to lead to a greater T-lymphocytes activation rather than estrogens that stimulate inhibition of T cells [21]. As regards the more frequent involvement of young individuals, it has been hypothesized that it could be due to a stronger and reactive immune response as compared to older patients (higher levels of TNF alpha and IFN gamma in the youth as compared to the older age) [21]. Nevertheless, our cohort included a not negligible number of adults, thus VAMPs cannot be considered almost exclusively affecting the pediatric population.

In agreement with prior studies (Table 3), myocarditis, myopericarditis and pericarditis mostly presented as paucisymptomatic forms (chest pain, fever and dyspnea the most frequent symptoms) with preserved left ventricular function, generally self-limiting and with resolution of signs of active inflammation at the CMR short-time follow-up [25, 26].

**Table 3:** Clinical and CMR data from the previous observational studies

	Jain SS et al., *Pediatrics* 2021;148	Das BB et al., *J Pediatr* 2021;238:26–32.e1	Dionne A et al., *JAMA Cardiol* 2021;6:1446	Puchalski M et al., *Int J Environ Res Public Health* 2022;19:3456	Truong DT et al., *Circulation* 2022;145:345–56	Amir G et al., *Pediatr Cardiol* 23.3.2022	Manfredi R et al., *Vaccines* 2022;10:169
Population characteristics							
Patient Population (n)	63	25	15	5	139	15	6
Age (mean ± SD or median [range])	15,6 ± 1,8	15 ± 1,5	15 [12–18]	16,6 ± 0,8	15,8 [1–20]	17 ± 1	17,5 ± 3,9
Sex (Males)	58 (92)	22 (88)	14 (93)	5 (100)	126 (91)	14 (93)	4 (66)
Vaccine							
Dose vaccine, % (I/II/Booster)	0/98/0	0/88/0	7/93/0	60/40/0	8,6/91,4/0	6/86/6	0/100/0
mRNA-based	63 (100)	25 (100)	15 (100)	5 (100)	136 (97,8)	15 (100)	6 (100)
Type, % (Comirnaty/Spikevax/Others)	94/6/0	100/0/0	100/0/0	100/0/0	94,2/3,6/2,1	100/0/0	66,7/33,3/0
Dose-to-onset interval, days (mean ± SD or median [range])	2,1 ± 1,3	3,1 ± 3,6	2,8 ± 1,3	2 [2–23]	2 [0–22]	4,4 ± 6,7	2,7 ± 1,2
Clinical presentation							
Chest Pain	63 (100)	25 (100)	15 (100)	5 (100)	138 (99)	15 (100)	/
Fever	28 (44)	6 (25)	10 (67)	4 (80)	43 (30,9)	4 (26)	5 (83)
Other symptoms [e,g, palpitation, dyspnea, myalgia]	46 (73)	4 (16)	8 (53)	0	64 (46)	0	0
Troponin increase	63 (100)	25 (100)	15 (100)	5 (100)	139 (100)	14 (93)	6 (100)
ECG anomalies	44 (70)	21 (84)	9 (60)	5 (100)	97 (69,8)	13 (86)	1 (16)
ECG arrhythmias	4 (6)	5 (20)	1 (7)	0	14 (10)	0	1 (16)
Lenght Hospitalization (days, mean ± SD or [range])	3,0 ± 1,4	2 [0–7]	2,6 ± 1,2	/	2 [0–10]	/	7 ± 2
Cardiac magnetic resonance							
Number CMR exams	56	16	15	5	97	15	6
Onset-to-CMR interval, days (mean ± SD or [range])	4,9 ± 2,3	/	3 [1–7]	14,10 ± 11,4	5 [1–88]	28 ± 21	3,5 [3, 4]
LLC positivity	49 (88)	6 (37)	3 (20)	5 (100)	49 (50,5)	4 (26)	4 (67)
Reduced LV-EF	14 (25)	/	3 (20)	0	0	/	0
LGE presence	49 (88)	15 (94)	12 (80)	5 (100)	14 (80)	14 (93)	4 (67)
Pericardial effusion or inflammation	/	3 (19)	/	0	/	3 (20)	2 (33)
Follow-up							
Clinical symptoms	7 (13)	0	4 (27)	0	/	0	0
Troponin increase	3 (11)	/	3 (20)	0	/	/	0
ECG anomalies	12 (20)	/	4 (23)	1 (20)	/	/	0
FU CMR (n)	2	0	10	5	0	9	6
Reduced LV-EF	0	/	0	0	/	1 (11)	0
LGE presence	2 (100)	/	10 (100)	5 (100)	/	7 (77)	0

Although in other series rare occurrence of intensive care unit admission (8.7%) and a mortality rate of 1.4% were reported [21], in our cohort no patient had severe or life-threatening conditions.

VAMPs seem to have a more benign prognosis as compared to myocarditis associated to COVID-19 with almost complete resolution of symptoms at short-term follow-up [27, 28]. Patone et al. [27] estimated the risk of developing acute myocarditis after the first or second dose of adenovirus or mRNA vaccination to be 1–10 per million, approximately; on the other hand, the risk of myocarditis following SARS-CoV-2 infection is placed around 40 per million.

Moreover, myocarditis represents only one of the potential cardiovascular complications related to Sars-Cov-2 infection [29–31] which are associated to increased risk of in-hospital mortality and worse prognosis at one-year follow-up [32], confirming the advantages of immunization in preventing cardiovascular diseases as compared to the risks associated with SARS-CoV-2 infection.

In line with previous studies [19, 22, 26, 33], the majority of our patients revealed clinical signs and symptoms of cardiac involvement after the second dose of mRNA vaccines (45.5%), even though the number of patients with onset after the first dose was consistent (40.9%).

Our CMR findings confirm those from Fronza et al. [26], which reported a low rate of LV systolic dysfunction and regional wall motion abnormalities in VAMPs patients as well as the prevalence and distribution pattern of myocardial edema and LGE (predominantly subepicardial and mid-wall). Those authors also demonstrated that CMR findings in vaccine-related myocarditis were similar to other forms with different etiologies, but with milder myocardial impairment (higher LV and RV EF and less extensive LGE were found in VAMP) [26].

A large meta-analysis of 102 studies, including a total of 468 patients with clinically suspected myocarditis following COVID-19 vaccination [34], demonstrated that left ventricular dysfunction is uncommon at clinical onset (LV-EF < 50%: 9.2%) and CMR signs of myocardial inflammation are frequent with rates very similar to those of our series (elevated nT1: 74.5%, T2 weighted or T2 STIR or T2 mapping abnormality: 81.9%; presence of LGE: 94%). Interestingly, pericardial enhancement was found in 32.8% of patients, confirming that pericardial involvement is a quite common feature in this condition.

The novel aspect of our study consists in the comparison between CMR acquired at baseline and at short-term follow-up. Most of the patients improved their LV and RV systolic function at FU and signs of active inflammation were still evident in 8/29 patients. Residual LGE was found in 26/29, reflecting the fibrotic evolution of myocardial damage, which has been associated with an increased risk of major cardiovascular events [35, 36]. It is known that in patients with viral myocarditis, the presence of LGE with anterior or septal midwall LGE is correlated to a greater mortality rate as compared with other LGE distribution patterns or with the complete absence of LGE [37]. In our study population, most of the patients showed an infero-lateral LGE location, suggesting a more favorable outcome; but the real prognostic impact of LGE in this population should be investigated with long-term follow-up studies with larger cohorts.

At short-term CMR-FU, VAMPs showed similar features as compared to viral myocarditis: persistence of LGE, progressive resolution of myocardial edema on T2 weighted images [38] and decrease of T1 and T2 mapping values [38]. As regards LVEF, a study by Ammirati et al. [39] conducted on 76 patients with classical acute myocarditis revealed the increase of systolic function at a median follow-up of 148 days in whom had a baseline EF < 55% [39].

The similarity of CMR features between VAMPs and other forms of myocarditis likely reflects analogous pathophysiological mechanisms. Several pathways of myocardial injury have been hypothesized following COVID-19 vaccination. Firstly, the so-called “molecular mimicry” theory, in which the immune cross-reactivity between the viral antigen and myocardial proteins (e.g. alpha-myosin) is induced [19, 20], that could justify the prevalence of acute myocarditis after the second dose [24]. Another hypothesis considers the development of an inflammatory response against the mRNA detected as an antigen by the immune system [21]. Finally, the viral surface protein seems to interact with angiotensin converting enzyme 2 receptors, stimulating the immune system activation and cardiac sensitivity [22].

General agreement converges on a transient dysregulated immune response, also supported by the evidence at endomyocardial biopsy of mixed inflammatory infiltrates with acute lymphocytic myocarditis [24] or degranulated eosinophils consistent with a pattern of hypersensitivity myocarditis [40].

### Study limitations

The study is retrospective and the analysis collects data from several hospitals, where both clinical assessment and diagnostic examinations were subject to the local physicians’ decisions. CMR were performed with different scanners and protocols. In particular, T1 or T2 mapping sequences were not always available or performed and this could have affected diagnostic performance of CMR in those centers, resulting in lower sensitivity for cases with subtle or diffuse myocardial damage. Moreover, our cohort included only patients with CMR findings consistent with diagnosis of myocarditis and/or pericarditis, therefore it is likely that cases of minimal vaccine-related myocardial injury, not detectable by CMR, could have been excluded from the enrollment. Furthermore, in patients scanned at follow-up without mapping sequences, we cannot exclude that mild active inflammation persisted even in the absence of areas of hyperintensity on edema-weighted sequences. Another limitation is the non-negligible number of patients who did not perform clinical (13/57, 22.8%) or CMR (15/44, 34%) follow-up, even though the number of subjects who completed the assessment was sufficient to perform the analysis. Histological confirmation, actually still the gold standard for diagnosing acute myocarditis, was not obtained in our entire patients’ population and therefore diagnosis of myocarditis was based on clinical and CMR findings in most of the cases.

The definition of VAMPs relied on the temporal association between COVID-19 vaccination and the onset of symptoms and we are not able to exclude other causes of myocarditis or pericarditis.

## Conclusion

Acute myocarditis, pericarditis or myopericarditis following COVID-19 vaccination are generally characterized by mild clinical presentation with typical CMR features of myocardial and/or pericardial inflammation. Short-term follow-up demonstrated self-limiting course and resolution of CMR signs of active inflammation in most of the cases. Further studies with larger case series and longer follow-up are required to better understand the characteristics of this syndrome, long-term outcomes and to depict its peculiarities with respect to the other forms of myocarditis and pericarditis.

## Supplementary Information

Below is the link to the electronic supplementary material.

Supplementary file1 (PDF 50 KB)

## Abbreviations

CMR: Cardiac magnetic resonance
FU: Follow-up
LGE: Late gadolinium enhancement
LV: Left ventricle
LVEF: Left ventricular ejection fraction
RV: Right ventricle
VAMP: Vaccine-associated myocarditis and pericarditis
