# Supplementary material for: Short term outcome of myocarditis and pericarditis following COVID-19 vaccines: a cardiac magnetic resonance imaging study
Source: Int J Cardiovasc Imaging. 2023 Mar 13;39(5):1031–43. doi: 10.1007/s10554-023-02799-w (PMC10009344; doi:10.1007/s10554-023-02799-w)
Supplement: Supplementary file 1 — Supplementary file1 (PDF 50 KB) [file 10554_2023_2799_MOESM1_ESM.pdf]

Site	Magnetic resonance scanner brand, model	Field strenght	Local protocol	Contrast medium
Ospedale Molinette, Torino, Italy	Philips, Achieva	1.5T	Cine-SSFP short and long axis (2Ch, 4Ch, 3Ch) + T2w STIR short and long axis + T1 mapping pre- and post-Gd (ShMOLLI) and T2 mapping (mGRASE) (3 slices on short axis) + LGE (IR-GRE) short and long axis (10 min after injection)	Gadobutrol 0.15 mmol/kg
Azienda Ospedaliero-Universitaria di Cagliari, Cagliari, Italy	Philips, Achieva D-STREAM	1.5T	Cine-SSFP short and long axis (2Ch, 4Ch, 3Ch) + T2w STIR short and long axis + T1 mapping pre- and post-Gd (ShMOLLI) and T2 mapping (mGRASE) (3 slices on short axis) + LGE (PSIR) short and long axis (10-12 min after injection)	Gadobutrol 0.15 mmol/kg
Ospedale del Mare di Napoli, Napoli, Italy	Siemens, Magnetom Amira; Siemens, Magnetom Sola	1.5T	Axial T2w TRUFI on thorax + Cine-SSFP short and long axis (2Ch, 4Ch, 3Ch) + T2w STIR (TIRM) short axis + EGE (PSIR) (3 min after injection) + LGE (PSIR) (short and long axis) (10 min after injection)	Gadobutrol 0.1 mmol/kg<br>or Gadoterate meglumine 0.2 mmol/kg
IRCCS Policlinico San Donato, San Donato Milanese (MI), Italy	Siemens, Magnetom Aera	1.5T	Cine-SSFP short and long axis (2Ch, 4Ch, 3Ch) + T2w STIR (TIRM) short axis + T2 mapping (T2p-SSFP) (3 slices on short axis) + LGE (IR-GRE) short and long axis (10-12 min after injection)	Gadobutrol 0.1 mmol/kg
Fondazione Toscana Gabriele Monasterio, Pisa, Italy	GE, Signa Artist	1.5T	Cine-SSFP short and long axis (2Ch, 4Ch, 3Ch) + T2w STIR short and long axis + T1 mapping pre- and post-Gd (ShMOLLI) (3 slices on short axis) + LGE (IR-GRE) short and long axis (10 min after injection)	Gadoterate meglumine 0.2 mmol/Kg
Ospedale SS. Annunziata, Chieti, Italy	Philips, Ingenia	3T	Cine-SSFP short and long axis (2Ch, 4Ch, 3Ch) + T2w STIR short axis + T1 mapping (MOLLI) and T2 mapping (GRASE) (3 slices on short axis) + EGE (PSIR) (3 min after injection) + LGE (PSIR) (short and long axis) (10 min after injection)	Dotarem 0.15 mmol/kg
Ospedale Regionale San Salvatore, L'Aquila, Italy	GE, Signa Horizon	1.5T	Cine-SSFP short and long axis (2Ch, 4Ch, 3Ch) + T2w STIR short and long axis + LGE (IR-GRE) short and long axis (10 min after injection)	Gadobutrol 0.1 mmol/kg
Ospedale Fatebenefratelli - Isola Tiberina, Roma, Italy	Philips, Achieva D-STREAM	1.5T	Cine-SSFP short and long axis (2Ch, 4Ch, 3Ch) + T2w STIR short and long axis + T1 mapping pre- and post-Gd (MOLLI) and T2 mapping (mGRASE) (3 slices on short axis) + LGE (PSIR) short and long axis (10-15 min after injection)	Gadobutrol 0.1 mmol/kg
Azienda Ospedaliero Universitaria Careggi, Firenze, Italy	Siemens, Magnetom Aera	1.5T	Axial, Coronal, Sagittal T2w TRUFI on thorax + Cine-SSFP short axis, long axis (2Ch, 4Ch, 3Ch), LVOT (cor + sag) and RVOT (cor + sag) + T2w STIR short and long axis + T1 mapping pre- and post-Gd (MOLLI) (3 slices on short axis) + T2 mapping (T2p-SSFP) (3 slices on short axis) LGE (PSIR) short and long axis (10 min after injection)	Gadobutrol 0.1 mmol/kg
IRCCS Ospedale San Raffaele, Milano, Italy	Philips, Ingenia	1.5T	Cine-SSFP short and long axis (2Ch, 4Ch, 3Ch) + T2w STIR short axis + T1 mapping pre- and post-Gd (MOLLI) and T2 mapping (GRASE) (3 slices on short axis) + LGE (PSIR) short and long axis (8-10 min after injection)	Gadobutrol 0.1 mmol/kg
IRCCS Humanitas Research Hospital, Rozzano (MI), Italy	Siemens, Magnetom Aera	1.5T	Cine-SSFP short and long axis (2Ch, 4Ch, 3Ch) + T2w STIR short and long axis + T1 mapping pre- and post-Gd (ShMOLLI) and T2 mapping (T2p-SSFP) (3 slices on short axis) + LGE (PSIR) short and long axis (8-10 min after injection)	Gadobutrol 0.1 mmol/kg
IRCCS Policlinico Sant'Orsola-Malpighi, Bologna, Italy	Philips, Ingenia	1.5T	Cine-SSFP long axis (2Ch, 4Ch, 3Ch) + T2w STIR or SPIR short and long axis (AC stack and 4ch) + T1 mapping native (MOLLI) and T2 mapping (GraSE) (3 slices on short axis) + Cine-SSFP short axis (AC stack) + LGE (PSIR) short axis stack and long axis (4ch, 2ch) (8-10 min after injection)	Gadobutrol 0.1 mmol/kg
Policlinico Umberto I, Roma, Italy	Siemens, Avanto	1.5 T	Cine-SSFP short and long axis (2Ch, 4Ch, 3Ch) + T2w STIR short and long axis + T1 mapping pre- and post-Gd (MOLLI) and T2 mapping (T2-3pt-GRE) (3 slices on short axis) + LGE (PSIR) short and long axis (8-10 min after injection)	Gadobutrol 0.1 mmol/kg
